# Assessing the potential effects and cost-effectiveness of programmatic herpes zoster vaccination of elderly in the Netherlands

**DOI:** 10.1186/1472-6963-10-237

**Published:** 2010-08-13

**Authors:** Alies van Lier, Albert Jan van Hoek, Wim Opstelten, Hein J Boot, Hester E de Melker

**Affiliations:** 1 Department of Epidemiology and Surveillance, Centre for Infectious Disease Control, National Institute for Public Health and the Environment, Bilthoven, The Netherlands; 2 Modelling and Economics Unit, Health Protection Agency, Centre for Infections, London, UK; 3 Julius Center for Health Sciences and Primary Care, University Medical Center Utrecht, Utrecht, The Netherlands; 4 Laboratory for Infectious Disease Diagnostics and Screening, Centre for Infectious Disease Control, National Institute for Public Health and the Environment, Bilthoven, The Netherlands

## Abstract

**Background:**

Herpes zoster (HZ) is a painful disease affecting a considerable part of the elderly. Programmatic HZ vaccination of elderly people may considerably reduce HZ morbidity and its related costs, but the extent of these effects is unknown. In this article, the potential effects and cost-effectiveness of programmatic HZ vaccination of elderly in the Netherlands have been assessed according to a framework that was developed to support evidence-based decision making regarding inclusion of new vaccines in the Dutch National Immunization Program.

**Methods:**

An analytical framework was used combining a checklist, which structured relevant data on the vaccine, pathogen and disease, and a cost-effectiveness analysis. The cost-effectiveness analysis was performed from a societal perspective, using a Markov-cohort-model. Simultaneous vaccination with influenza was assumed.

**Results:**

Due to the combination of waning immunity after vaccination and a reduced efficacy of vaccination at high ages, the most optimal cost-effectiveness ratio (€21716 per QALY) for HZ vaccination in the Netherlands was found for 70-year olds. This estimated ratio is just above the socially accepted threshold in the Netherlands of €20000 per QALY. If additional reduction of postherpetic neuralgia was included, the cost-effectiveness ratio improved (~€10000 per QALY) but uncertainty for this scenario is high.

**Conclusions:**

Vaccination against HZ at the age of 70 years seems marginally cost-effective in the Netherlands. Due to limited vaccine efficacy a considerable part of the disease burden caused by HZ will remain, even with optimal acceptance of programmatic vaccination.

## Background

The varicella-zoster virus (VZV) causes varicella (chicken pox) as well as herpes zoster (HZ, shingles). Varicella is the primary infection, whereas HZ is caused by reactivation of latent VZV in sensory nerve ganglia. HZ is characterized by a painful localized vesicular rash. The most common complication of HZ is postherpetic neuralgia (PHN), a chronic pain condition that can last for months or even years. In contrast to varicella, which is mainly a childhood disease, HZ predominantly affects older adults [1]. Presently, a vaccine to prevent HZ is available [2]. In this article, we present an assessment of the potential effects of programmatic HZ vaccination of elderly in the Netherlands. Fur this purpose we used a framework that was developed to support evidence-based decision making regarding inclusion of new vaccines in the Dutch National Immunization Program (NIP). This framework consists of a checklist that structures all relevant data on vaccine, pathogen and disease [3]. These data, presented in the Background section, are input to a cost-effectiveness analysis that is presented in the Methods and Results section.

### Vaccine

#### Available vaccines and indications

Only one vaccine (ZOSTAVAX^®^; SP-MSD) has been registered for the prevention of HZ. This live attenuated vaccine is manufactured by the same process as the chicken pox vaccine VARIVAX^® ^but has a higher viral load per dose [2]. The vaccine has been registered in the EU as a single dose vaccine for the prevention of HZ and PHN among people aged 50 years or older.

#### Vaccine efficacy

Natural protection against HZ may occur by exogenous boosting (due to circulating VZV in the population) or endogenous boosting (through subclinical reactivation of latent VZV). Although the mechanism of latency is not fully understood, there is strong evidence that the risk of developing HZ is linked to a decline in VZV-specific cell-mediated immunity (CMI) [1,4]. The functional mechanism of the vaccine is to boost this specific CMI [2].

**Figure 1:**
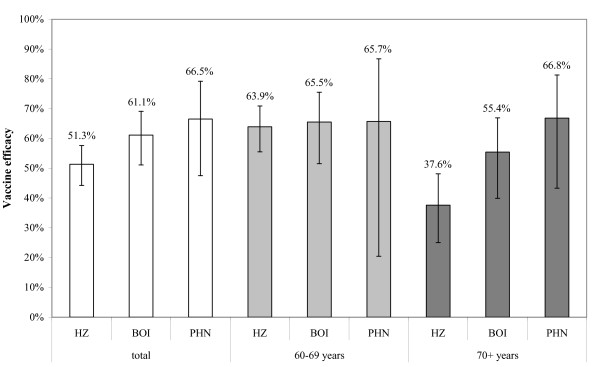
**Overview of the vaccine efficacy with respect to the incidence of herpes zoster (HZ), burden of illness (BOI) and incidence of postherpetic neuralgia (PHN) by age-group**. (source: Oxman MN, Levin MJ, Johnson GR *et al. *A vaccine to prevent herpes zoster and postherpetic neuralgia in older adults. N Engl J Med 2005; 352(22):2271-84).

The efficacy of the vaccine was assessed in a large randomized placebo-controlled trial. There was a reduction of 51.3% in the incidence of HZ, 61.1% in the burden of illness (BOI) and 66.5% in the incidence of PHN [5]. The vaccine appeared less effective in the older age group (70+ years) compared to the younger age group (60-69 years) (Figure 1), indicating that the effect of vaccination is age dependent [5]. The long term efficacy of the vaccine is unknown (mean follow-up duration so far was three years), but the immunity seems to decrease over time after vaccination [2].

#### Contra-indications and adverse events following vaccination

Since the vaccine consists of live-attenuated virus, it should not be used in immunocompromised people, people with active untreated tuberculosis or in pregnant women [2].

Adverse events at the injection site occurred more frequently in the vaccine group (48.3%) compared to the placebo group (16.6%), but most of them were mild. Furthermore, vaccine-related systemic adverse events occurred more frequently in the vaccine group than in the placebo group (6.3% vs 4.9%) [5].

#### Factors affecting successful implementation

So far, influenza vaccination is the only generally advised vaccination for elderly in the Netherlands. The general practitioner (GP) invites all people aged 60 years or older annually for this vaccination, which has a high coverage (in 2008/2009 76.9%) [6]. HZ and influenza vaccine given concomitantly are well tolerated [7]. Furthermore, antibody responses were similar compared to sequential vaccination. A recent study, however, showed that among community-dwelling elderly to whom both influenza and HZ vaccination were offered within an existing influenza vaccination program, only 39% accepted HZ vaccination, whereas 76% accepted influenza vaccination [8]. Determinants of non-compliance with additional HZ vaccination were: perceived lack of recommendation by the GP, unwillingness to comply with the doctor's advice, perception of low risk of contracting HZ, perception of short pain duration of HZ and the opinion that vaccinations weaken one's natural defenses [8]. Other studies also found that a recommendation by the GP is a major determining factor of accepting vaccination in this age group [9,10]. An international survey pointed out that the understanding of the risk of developing HZ, its symptoms, complications and treatment among adults ≥55 years of age is very limited [11]. Moreover, in the United States the lack of patient awareness and physician recommendation were pointed to be barriers to HZ vaccine uptake [10].

#### Herd immunity

The transmission of VZV resulting from patients with HZ is very low in comparison to varicella [1]: therefore no herd immunity effects are to be expected. Reaching a high vaccination coverage is therefore not important, unlike for most other vaccinations. HZ vaccination will only give benefit on individual level.

### Pathogen

#### Pathogenicity

VZV-seroprevalence in the Netherlands approaches 100% from seven years onwards [12]. In HIV-infected persons the risk of HZ and its recurrence is increased (12-17 fold) [1]. Intercurrent infection with viruses that can alter CMI responses (such as Epstein-Barr virus and cytomegalovirus) also influences the risk of developing HZ [4].

#### Infectiveness and transmissibility

HZ is not transmitted directly; it is a reactivation of VZV that remains latent in sensory nerve ganglia after primary VZV-infection. The herpes lesions are contagious for non-immune persons (until the lesions have crusted) and can lead to varicella [1]. Subclinical reactivation of the VZV virus is possible but the frequency of occurrence is unknown [4]. In immunocompetent individuals, the frequency of recurrent HZ is low (1.7-5.2%) [13].

#### Antigenic variation

The VZV genome is extremely stable. So far, seven distinct genotypes of the wild-type VZV have been distinguished with a different geographic distribution, but all belong to the same serotype. No evidence for recombination among wild-type VZV-strains has yet been found [14]. Although recombination events could theoretically alter the virulence of circulating VZV strains [15], the impact of such events would probably be very small. Ecological consequences after implementation of vaccination are not expected. VZV is an exclusively human pathogen. Both the vaccine strain and the wild-type VZV establish a latent infection. Furthermore, interaction or competition with other alpha-herpes viruses like HSV-1 and HSV-2 has not been described for VZV [16].

### Burden of disease

#### Risk factors for herpes zoster

It is estimated that 23-30% of the population in Europe will develop HZ during their lifetime; approximately 50% of all people reaching the age of 85 years will have experienced HZ [13]. Prior infection with VZV, either with wild-type or vaccine virus, is a prerequisite for developing HZ. The vaccine virus may have less opportunity to reactivate than does wild-type VZV [4]. The vaccine virus usually does not cause viremia or skin infection, factors that are both likely to enhance the development of HZ [17].

The incidence of HZ increases with age, which is attributed to the natural process of age-related immunosenescence. Furthermore, the incidence is higher among people with immunity attenuating diseases or medication [1,4,18]. Other possible risk factors that have been suggested are physical trauma at the involved dermatome, psychological stress, changes in mental health, depression, white race and intercurrent infection with viruses that can alter CMI responses [1,4,18]. Some studies show also higher incidence rates in women, even after correction for higher average age and health care seeking behavior [18,19]. VZV-infection in utero or shortly after birth has been found to be a risk factor for (childhood) HZ [1,4,18]. PHN is more likely to occur in older HZ patients and in HZ patients with severe pain or rash during the acute phase [4,18,20].

#### Consequences of herpes zoster

HZ begins with a prodrome, during which abnormal skin sensations and pain of varying severity are the most common symptoms, followed by a vesicular rash. This rash is typically unilateral, does not cross the mid-line, normally involves a single dermatome, is usually accompanied by acute pain and lasts for 7-10 days or longer. PHN, a persistent pain after resolution of the rash, is the most important complication of HZ and can last for several years [1,4]. Therapeutical options for HZ and PHN are scarce. About half of the patients with PHN will benefit from therapy with only partial relief [4]. The quality of life during HZ is influenced by the severity and duration of the acute and chronic pain that can affect physical, psychological, social and functional domains [1,4].

#### Alternative preventive measures

There are no direct alternatives to prevent HZ. Childhood vaccination against varicella might reduce the HZ incidence on the long term, because the vaccine strain is less likely to cause HZ than the wild-type. However, reduced VZV transmission due to varicella vaccination will diminish exogenous exposure (boosting), which might lead to an increase in the incidence of HZ in the mid-term (the first 30-50 years) [21]. Studies monitoring the incidence of HZ in the US, where universal vaccination against varicella was introduced in 1995, have shown inconsistent findings at this point. Two studies did not show an increase in overall incidence [22,23], whereas three others demonstrated a rise [24-26].

## Methods

### Data sources

#### GP consultations, hospitalizations and deaths

Most HZ patients will consult their GP as it is a painful condition. Age-specific incidence rates for the period 2002-2007 were derived from the Netherlands Information Network of General Practice (LINH) [27]. A correction was made for false positives (10%; 7.9-12.4%[28]) and immunocompromised people (5%[28]), since both groups will not benefit from vaccination. A linear regression was plotted on the HZ incidence of the separate years 2002-2007.

Hospitalization data (ICD-9 code 053) were taken from the National Medical Register (LMR) for the period 2000-2007. Only admissions with HZ as main diagnosis were included because these admissions represent cases that are preventable by vaccination. The incidence of clinical admissions was rather stable in the period 2000-2007. However, the incidence of admissions for one day decreased from 7.5 per 100000 in 2002 to 4.0 per 100000 in 2007 [29]. Therefore, an alternative scenario was included in which the daytime hospital visits were excluded. The distribution used in the probabilistic sensitivity analysis is listed in Additional file 1.

Mortality data (ICD-10 code B02 and G530) for the period 2000-2007 were derived from Statistics Netherlands (CBS). Only deaths with HZ as primary cause of death were included in the base case scenario. An alternative scenario without prevention of death was also included since it is likely that death is not caused directly by HZ.

#### Pain, incidence of PHN and quality-adjusted life-year (QALY) loss

The duration of pain by severity and age, and subsequently the QALY loss due to HZ, was estimated by Van Hoek *et al *[28] and applied to the Dutch situation. For clarity, this does include PHN which was defined as the presence of clinical relevant pain after three months. In the model QALY loss after onset was modeled based on the duration spent in clinical relevant or mild pain [28] instead of using a fixed percentage developing PHN.

### Vaccine parameters

We used the vaccine efficacy as estimated by Van Hoek *et al *[28]. The vaccine efficacy was split into two parameters, a take (initial vaccine efficacy) and waning (reduction of protection over time) and those two parameters were estimated on the data from the initial clinical trial. The base case waning was only 7.5 years and was estimated to be between 3.6 to 100 years, with an age dependent take. In the sensitivity analyses the effects of a longer and shorter duration of protection were calculated. Based on the coverage for influenza vaccination in the Netherlands, we assumed a vaccine coverage of 75%.

The different protection of the vaccine against the three endpoints (Figure 1) as measured in the clinical trial was simulated by three different scenarios. In the scenario based on the reduction of HZ only, the reduction of HZ and subsequent QALY loss was included. In the scenario describing the reduction of BOI, a reduction of QALY loss for the first 6 months in people with disease was included above the reduction in HZ cases (this is because the vaccine reduces disease severity in cases where HZ occurs in spite of vaccination). For the reduction of PHN (only applicable above the age of 70 years) the number of people in clinically relevant pain was decreased by the specific vaccine efficacy [28,30]. If not mentioned otherwise, presented numbers are based on the protection against BOI (base case), the main endpoint in the clinical trial.

### Cost data

All costs are presented in 2008 Euros: costs in previous years were deflated with the consumer price index according to CBS. To assess the costs of an average HZ or PHN case, the in depth patient data as collected within the PINE study was used [31]. Patients were considered to suffer from PHN if they had a pain level of at least 25 (on a scale of 0-100) at three months after onset. The cost assumptions that were used in this assessment are described in Additional file 2.

#### Direct costs of disease

The major costs involved in HZ are the prescription of antivirals and repetitive GP visits for PHN patients. In the PINE study, detailed information on GP consultations, medication and additional use of health services due to HZ was available for the first 6 months of the study (Additional file 2). Based on those findings the average total costs per patient of GP consultations and drug use is €72.05 (€66.90 - €77.20) in case of HZ and €101.10 (€81.72 - €120.70) for PHN based on the first 6 months of the study. Because the duration of PHN can be longer, these costs were doubled: €201.91 (€163.30 - €241.15). Confidence intervals of the mean price (95%) were acquired by bootstrapping.

#### Indirect costs of disease

Indirect costs were considered for estimated work loss till the age of 65. Data on work loss due to HZ is scarce and the participation in the workforce is not high in the age group 60+. A questionnaire among 65 HZ patients in the UK [32] showed that 29 patients were employed, with an average working loss of 10.1 days (SD of mean 1.82). According to CBS, participation in the work force (in 2006) was only 20.8% in the age group 60-65. The number of hours of labour per week is 32 or 6.4 per day with a payment of €24.10 per hour. With a correction for participation in the workforce this is an average of €32.04 lost per day or €324 for the total work loss for someone in the age group 60-65.

#### Cost of the vaccine and the vaccination program

Because the HZ vaccine is not yet available in the Netherlands, the Dutch price is unknown. The official retail price of the HZ vaccine in the US is $153.93 or €110 (Pack 10-Vial; January 2009). However, in case of introduction in the NIP, the CDC price of $107.67 or approximately €77 (January 2009) seems more applicable. In the sensitivity analyses the effect of lower vaccine prices was calculated.

Based on experience with the introduction of the pneumococcal vaccine in the Netherlands in 2006, the once-only costs (not included in the cost-effectiveness model) are estimated to be €0.3 million and include costs for education of GPs, developing information material (invitation letter, flyer, publicity campaign, website), adjustment of software for registration and monitoring, and administration. In case of implementing HZ vaccination within the current influenza vaccination program and assuming a vaccination coverage of 75%, the estimated yearly administration costs range from ~€14.7 million for vaccinating people at the age of 60 to ~€4.9 million for vaccination at the age of 80. This includes compensating vaccination personnel (€4.80 per application, this is half the influenza tariff) and coordination costs (€1.65 per application). In the sensitivity analyses the effect of higher applications costs (€9.60 instead of €4.80 per vaccination) was calculated. Monitoring of adverse events can be included in the already existing passive surveillance system, for which the total costs are estimated to be €0.4 million per year. Vaccine effectiveness, reflected by the reduction of the incidence of HZ, PHN and related hospitalizations, could be monitored using GP and hospitalization statistics.

### Cost-effectiveness model

The cost-effectiveness analysis was performed from a societal perspective. The incremental cost-effectiveness ratio (ICER) was used to compare the quality of adjusted life years gained with the net costs of programmatic HZ vaccination (compared to no-vaccination). The prevented number of cases, costs, QALYs and the Incremental cost-effectiveness ratio (ICER) were calculated at different ages: 60, 65, 70, 75 and 80 years. According to the Dutch guidelines for health technology assessment, future costs and effects of vaccination were discounted with 4% and 1.5%, respectively.

A Markov-cohort-model was set up in Excel (Microsoft, USA) and univariate and probabilistic sensitivity analysis were performed with @Risk (Palisade, USA). The same model was used in a cost-effectiveness model for HZ vaccination in England and Wales [28]. The effect of different assumptions regarding the duration of protection of the vaccine, discount ratio, prevention of death, vaccine price, application costs and hospital daycare were investigated in the sensitivity analyses.

## Results

### Current burden of disease

**Figure 2:**
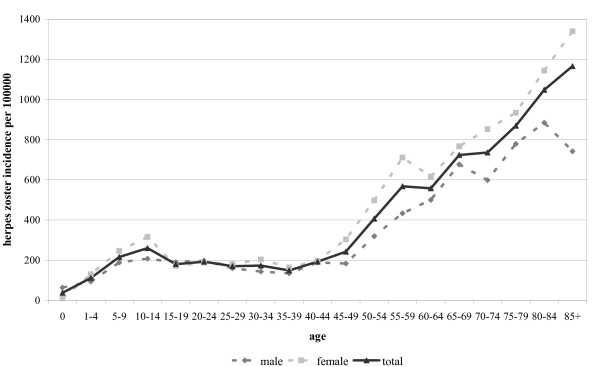
**Age-specific average annual incidence of GP-consultations due to herpes zoster per 100000 by sex 2002-2007**. (source: Verheij RA, van Dijk CE, Abrahamse H *et al. *Netherlands Information Network of General Practice (LINH): Facts and figures on GP care in the Netherlands. Utrecht/Nijmegen: NIVEL/WOK, 2008).

For the Netherlands, the average annual incidence of HZ based on GP consultations was 332 (range 310-370) per 100000 in the period 2002-2007. The incidence increases with age (Figure 2) [27]. The linear regression that was plotted on the HZ incidence of the separate years 2002-2007 predicted an incidence of 509 (394 - 626) per 100000 at the age of 60 and going up with 22 (17.1 - 27.0) per year.

**Figure 3:**
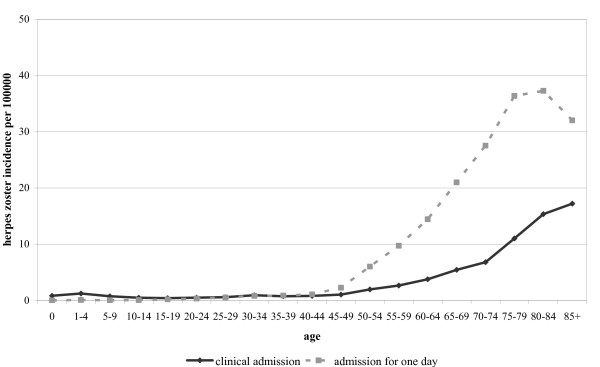
**Age-specific average annual incidence of hospital admissions due to main diagnosis herpes zoster per 100000 2000-2007**. (source: Prismant. National Medical Registration. Utrecht: Prismant, 2000-2007).

The average annual incidence of *clinical *hospital admissions due to *main *diagnosis HZ in the period 2000-2007 was 2.3 (range 2.0-2.7) per 100000 (when including *side *diagnosis HZ too, the total incidence was 4.7 (range 4.0-5.1) per 100000). In the same period, another 6.3 (range 4.0-7.5) hospital admissions *for one day *due to *main *diagnosis HZ were registered per 100000. The incidence of hospital admissions also increases with age (Figure 3). In the period 2000-2007 on average 18 deaths (range 13-26) with HZ as primary cause of death were registered annually. Most deaths occurred among people aged 75 years and older (92%).

The burden of disease in the Netherlands is estimated to be at highest in a cohort of 60 year olds (a loss of 3024 QALYs, discounted) and at lowest in a cohort of 80 year olds (a loss of 1060 QALYs, discounted) (Table 1). The ratio of QALY loss per HZ case (discounted), however, increases by age towards a maximum at the age of 80. Therefore the relative burden of disease is the highest at the age of 80. The estimated total costs for HZ for the group 60 year olds are almost €3.5 million per year; this is including an estimated €1.2 million of indirect costs. Although the estimated total costs for the group 80 year olds are lower (€0.8 million per year), the cost per HZ case in this age-group is higher than for 60 year olds (€177.79 versus €128.86) (Table 1).

**Table 1:** Absolute outcome and prevented cases for different ages at vaccination in the base case scenario

	*60 years*	*65 years*	*70 years*	*75 years*	*80 years*
***Before vaccination:***					
**Cases HZ**	26845	15513	11093	7630	4769
**Cases PHN**	4639	2936	2351	1857	1370
**Hospitalization**	320	205	163	128	89
**1 day visit hospital**	1102	683	515	363	210
**Deaths**	30.7	20.9	18.6	17.6	16.5
Direct costs*	€2217577	€1527388	€1306022	€1100313	€847884
Indirect costs*	€1241555	€0	€0	€0	€0
QALYs lost*	3024	2024	1703	1402	1060
					
***After vaccination:*** ***(75% coverage)***					
**Nr. of vaccinees**	175925	115943	94354	80712	58724
Vaccination costs**	€14680941	€9675443	€7873841	€6735416	€4900518
**Cases HZ**	22512	12496	9201	6277	4299
**Cases PHN**	4222	2581	2071	1603	1257
**Hospitalization**	292	178	141	107	81
**1 day visit hospital**	966	563	426	294	188
**Deaths**	30.5	20.6	18.1	16.4	15.4
Direct costs*	€1832919	€1219724	€1082777	€902727	€760458
Indirect costs*	€541068	€0	€0	€0	€0
QALYs lost*	2671	1724	1350	1133	921
					
***Prevented:***					
**Cases HZ**	4334	3017	1892	1352	471
**Cases PHN**	417	355	280	254	113
**Hospitalization**	28	27	21	20	9
**1 day visit hospital**	136	120	89	70	22
**Deaths**	0.2	0.3	0.5	1.2	1.1
Direct costs*	€384658	€307664	€223245	€197586	€87427
Indirect costs*	€700487	€0	€0	€0	€0
QALYs lost*	353	300	352	269	140

* Costs are discounted with 4% and QALYs with 1.5%

** Vaccination costs are based on a vaccine price of €77, application costs of €4.80 per vaccination and coordination costs of €1.65 per vaccination

### Effect of vaccination on cases and costs

Most cases (~4300) are prevented by vaccination at the age of 60. This number decreases to ~470 at the age of 80. The prevented number of deaths, however, increases by age at vaccination. From 0.2 prevented deaths by vaccination at 60 towards the maximum of 1.2 prevented deaths at the age of vaccination at 75 (Table 1).

By vaccinating people, costs regarding GP visits, prescription of antivirals and painkillers are prevented as well as hospitalization costs and costs due to work loss. For HZ vaccination the prevented costs are distributed equally between hospital costs and prevented cost generated in the GP practice. Prevented costs will reach a maximum of about €1085146 (or €384658 excluding indirect costs) for vaccinating people at the age of 60. The saved discounted costs, however, are low for each vaccinated person. Per vaccinee between €1.49 and €2.65 (or €6.17 including indirect costs) will be saved. Subsequently a vaccine price higher than this will have to be justified by preventing QALYs.

The absolute number of gained QALYs is the highest by vaccination at the age of 60 years with ~353, and the lowest by vaccination at the age of 80 years with a total gained of ~140. However this absolute number must be seen in the context of the number of people who have to be vaccinated to gain those QALYs. The number of people needed to be vaccinated to gain one QALY is a good proxy: the lowest number is 268 at the age of 70 years, the highest 498 at the age of 60 years.

### Cost-effectiveness of vaccination

**Figure 4:**
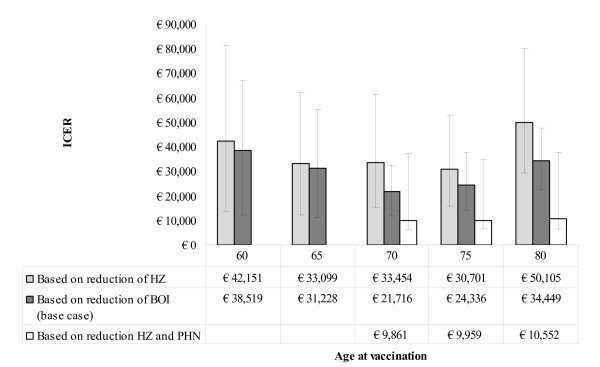
**Incremental cost-effectiveness ratio (ICER) for different scenarios and ages; indirect costs are included (loss of working hours, only relevant for vaccination at 60 years of age)**. The base case (dark grey) is including a lower QALY loss in the first 6 months of HZ among vaccinees, in the 'without additional effect' (light grey) this is not included. Error bars represent 95% confidence intervals and under the bars the relevant cost-effectiveness ratios are shown.

The information on the number of doses, vaccine efficacy, prevented costs and QALYs gained together is expressed in the cost-effectiveness ratio (Figure 4). Using the reduction of BOI as an endpoint the most optimal cost-effectiveness ratio is €21716 (95% CI: €11569 - €31870) for vaccination at the age of 70. The worst ratio is €38519 (95% CI: €12176 - €67158) for vaccination at the age of 60 under the same perspective (indirect costs included) or €40503 under the healthcare payer perspective (indirect costs excluded). This implies that vaccinating at the age of 70 results in the best value for money.

In the scenario with reduction of HZ cases only the cost-effectiveness ratio increases towards ~€33500 at the age of 70; using the scenario with reduction of PHN improves the cost-effectiveness to a ratio of ~€10000. Although the clinical trial showed a higher impact of vaccination on the BOI compared to the incidence of HZ, we want to mention that using BOI or PHN endpoints will be more sensitive towards the decisions made in the way the QALY loss due to HZ is currently modeled/estimated.

According to the sensitivity analyses (Table 2), changing assumptions regarding the discounting rate, vaccine price and duration of protection of the vaccine have the greatest impact on the ratio, especially with a longer duration of protection or a lower vaccine price the cost-effectiveness profile improves.

**Table 2:** Cost-effectiveness ratio under different circumstances and at different ages of vaccination

	60 years*	*65 years*	*70 year*	*75 years*	*80 years*
***Base case***	€38519	€31228	€21716	€24336	€34449
**No prevention of death**	€38901	€31489	€21910	€25020	€35930
**No daytime visits hospital**	€38540	€31251	€21731	€24351	€34458
**No discounting**	€33305	€27482	€18827	€21688	€31285
**Discounting 3.5%/3.5%**	€45313	€36210	€25647	€27874	€38725
**Vaccine price €60 per dose**	€30045	€24658	€17163	€19228	€27304
**Vaccine price €50 per dose**	€25061	€20793	€14485	€16224	€23100
Application costs €9.60**	€40911	€33083	€23002	€25778	€36466
Duration protection 4.8 years***	€61247	€48828	€27817	€32449	€42428
Duration protection 16.1 years***	€16954	€15031	€14030	€16013	€25953

* indirect costs included (loss of working hours, only relevant for vaccination at 60 years of age)

** full influenza tariff (instead of half the influenza tariff €4.80, that was used in the base case)

*** based on van Hoek AJ, Gay N, Melegaro A, Opstelten W, Edmunds WJ. Estimating the cost effectiveness of vaccination against herpes zoster in England and Wales. Vaccine 2009; 27(9):1454-67.

If a diagnostic test to determine immunity against VZV would become available in the future, a more targeted vaccination strategy could be implemented. Furthermore, people with a history of HZ could be excluded to save costs, as HZ does not frequently reoccur.

## Discussion

In view of the scarce therapeutic options for HZ and its sequelae the reduction of the risk of this disease by vaccination is an important development. Moreover, the HZ vaccine could be relevant because of the predicted temporary increase in the incidence of HZ after introducing childhood varicella vaccination [21]. HZ vaccination could prevent part of the disease burden of this often painful disease among elderly. However, the number of prevented GP-consultations, hospitalizations and deaths is relatively limited compared to other vaccine preventable diseases. In the decision process it is important to consider that the health gain that could be realized by HZ vaccination is in particular related to the reduction of (long term) pain; the number of life years gained is rather small. Furthermore, a considerable part of the disease burden caused by HZ will still remain despite programmatic vaccination since the vaccine efficacy is suboptimal. The indirect disease burden estimations might increase in future, if the recently reported increased risk of stroke after HZ is being confirmed in future research [33]. The relative low efficacy and the lack of knowledge on protection of the vaccine on the long term might be a problem for general acceptation of vaccination against HZ.

Offering HZ vaccination in combination with influenza could be a promising option. However, a previous Dutch study showed that the acceptance of HZ vaccination given simultaneously with influenza vaccination was only 39%, i.e. considerably lower compared to the vaccination coverage for influenza (76%) [8]. Insight into the degree of acceptance by the public is important, especially in the light of the recent experiences in the Netherlands with objection to introduction of the vaccine against human papillomavirus (HPV).

It will be difficult to make a decision on the targetgroup for HZ vaccination: the HZ incidence increases whereas the vaccine efficacy decreases with age. Based on the cost-effectiveness analysis (base case scenario), vaccinating at the age of 70 years would be the best option. However, the value of €21716 lies just above the socially accepted threshold in the Netherlands of €20000 per QALY. This implies that the cost-effectiveness profile is marginal, although this is not the first evaluation criterion for introduction of a new vaccine [34]. The scenario with additional reduction of PHN improves the cost-effectiveness to a ratio of ~€10000. However, this scenario has some major limitations. First, the definition of PHN as used in the clinical trial does not necessarily concern pain on the long term. Second, the effectiveness of the vaccine against PHN is not straightforward (extra effectiveness only above the age of 70 years) and has a high uncertainty. If the duration of protection turns out to be longer, the vaccination could be given at an earlier age which might improve the cost-effectiveness of the vaccine. Research on new vaccines with a higher vaccine efficacy, in particular at older age, is recommended.

There are several other estimations of the cost-effectiveness of HZ vaccination [28,35-39]. Most of those cost-effectiveness studies apply for the USA [35-37] and Canada [38,39] and one for the UK [28]. Because of differences between countries in health care costs and health care seeking behavior, direct comparisons are hard to make. Also, the assumptions regarding the vaccine price were different: $150 (€107) instead of the €77 assumed in this analysis (which is based on the lower price CDC pays for its vaccine). Nevertheless the majority of studies conclude that vaccination against HZ is cost-effective in their health care system, in contradiction with this study where it is marginally cost-effective. This difference can be mainly attributed to the differences in the threshold value used by the countries. Internationally the threshold of €20000 per QALY as used in the Netherlands is the lowest among the countries where a cost-effectiveness study was done. Moreover, the incidence among the elderly seems to be slightly lower in the Netherlands. Whether this is due to a slightly lower reportage in the Dutch general practice, due to uptake of patients in nursing homes (that are not included in the Dutch reporting system) or due to other factors is unknown.

## Conclusions

In conclusion, programmatic vaccination could reduce the burden of disease due to HZ considerably but is estimated to be marginally cost-effective even at the economically most attractive option, i.e. vaccination at the age of 70 years simultaneously with influenza vaccination. A final judgment on the cost-effectiveness will depend on price negotiations with the different parties involved. Even with vaccination at levels comparable to influenza vaccination, less than half of the disease burden caused by HZ will be prevented by vaccination, due to the relative low efficacy of the vaccine. It would be a challenge to reach high acceptance of vaccination despite the occurrence of HZ among vaccinees; involvement of the GP is essential.

While for many childhood vaccinations in addition to individual protection, indirect protection by herd immunity is offered, this does not hold for HZ. Making the public aware of the existence of a HZ vaccine (with its current limitations) that could be obtained individually is necessary, irrespective of the decision whether or not to implement programmatic vaccination.

## Abbreviations

BOI: burden of illness (a severity-by-duration measure of the total pain and discomfort associated with herpes zoster); CMI: cell-mediated immunity; GP: general practitioner; HZ: herpes zoster; ICER: Incremental cost-effectiveness ratio; NIP: National Immunization Program; PHN: postherpetic neuralgia (pain and discomfort associated with herpes zoster rated as 3 or more, on a scale ranging from 0 (no pain) to 10 (pain as bad as you can imagine), persisting or appearing more than 90 days after the onset of the herpes zoster rash); QALY: Quality Adjusted Life Year; VZV: varicella zoster virus.

## Competing interests

The authors declare that they have no competing interests.

## Authors' contributions

EAvL* drafted the manuscript, AJvH* performed the cost-effectiveness analysis and drafted the manuscript regarding this component. WO critically revised the manuscript. HJB initiated the design of the manuscript and helped to draft the manuscript. HEdM designed the vaccination evaluation framework and critically revised the manuscript. All authors read and approved the final manuscript.

* *These two authors contributed equally*

## Pre-publication history

The pre-publication history for this paper can be accessed here:

http://www.biomedcentral.com/1472-6963/10/237/prepub

## Supplementary Material

Additional file 1 **Used distribution regarding hospitalization in the sensitivity analyses**. In additional file 1 the distribution regarding hospitalization that was used in the sensitivity analyses is presented. Click here for file

Additional file 2 **Costs assumptions cost-effectiveness analysis**. In additional file 2 the assumptions regarding costs that were used in the cost-effectiveness analysis are presented. Click here for file
