# Supplementary material for: Assessing the potential effects and cost-effectiveness of programmatic herpes zoster vaccination of elderly in the Netherlands
Source: BMC Health Serv Res. 2010 Aug 13;10:237. doi: 10.1186/1472-6963-10-237 (PMC2928772; doi:10.1186/1472-6963-10-237)
Supplement: Additional file 1 — Used distribution regarding hospitalization in the sensitivity analyses. In additional file 1 the distribution regarding hospitalization that was used in the sensitivity analyses is presented. [file 1472-6963-10-237-S1.DOC]

**Additional file 1 Used distribution regarding hospitalization in the sensitivity analyses**

**1 day hospital visits due to herpes zoster in the hospital**

| *Age group* | *Percentage cases with a one day visit* | *Estimated number of cases in daycare 2007* | *Number of estimated HZ cases 2007* | *Distribution* |
| --- | --- | --- | --- | --- |
| 60-64 | 2.70 % | 149 | 5963 | Beta distribution |
| 65-69 | 3.49 % | 168 | 4850 | Beta distribution |
| 70-74 | 4.02 % | 184 | 4247 | Beta distribution |
| 75-79 | 4.57 % | 196 | 3641 | Beta distribution |
| 80-84 | 4.01 % | 137 | 2737 | Beta distribution |
| 85+ | 3.89 % | 90 | 2324 | Beta distribution |

Hospital admissions due to zoster

| *Age group* | *Percentage cases in hospital* | *Estimated number of cases in hospital 2007* | *Number of estimated HZ cases 2007* | *Distribution* |
| --- | --- | --- | --- | --- |
| 60-64 | 0.51 % | 28 | 5963 | Beta distribution |
| 65-69 | 0.78 % | 38 | 4850 | Beta distribution |
| 70-74 | 0.85 % | 39 | 4247 | Beta distribution |
| 75-79 | 1.12 % | 48 | 3641 | Beta distribution |
| 80-84 | 1.49 % | 51 | 2737 | Beta distribution |
| 85+ | 1.89 % | 44 | 2324 | Beta distribution |

Number of days in the hospital

| *Age group* | *Average days in hospital* | *Standard deviation* | *Distribution* |
| --- | --- | --- | --- |
| 60-64 | 8 | 1.24 | Normal distribution |
| 65-69 | 9 | 1.15 | Normal distribution |
| 70-74 | 9 | 1.21 | Normal distribution |
| 75-79 | 11 | 1.17 | Normal distribution |
| 80-84 | 14 | 1.57 | Normal distribution |
| 85+ | 17 | 2 | Normal distribution |
