# Supplementary material for: Assessing the potential effects and cost-effectiveness of programmatic herpes zoster vaccination of elderly in the Netherlands
Source: BMC Health Serv Res. 2010 Aug 13;10:237. doi: 10.1186/1472-6963-10-237 (PMC2928772; doi:10.1186/1472-6963-10-237)
Supplement: Additional file 2 — Costs assumptions cost-effectiveness analysis. In additional file 2 the assumptions regarding costs that were used in the cost-effectiveness analysis are presented. [file 1472-6963-10-237-S2.DOC]

**Additional file 2 Costs assumptions cost-effectiveness** analysis

| *Description* | *Costs* |
| --- | --- |
| GP consult | € 9.00 ^a^ |
| GP home visit | € 13.50 ^a^ |
| GP telephone consult | € 4.50 ^a^ |
| **Antivirals**: |  |
| Aciclovir (800mg; 35 tablets) | € 28.84 ^b^ |
| Famciclovir (500mg; 21 tablets) | €116.97 ^b^ |
| Valaciclovir (500mg; 42 tablets) | €103.90 ^b^ |
| **Pain medication** |  |
| Gabapentine (300mg; 30 tablets) | € 10.00 ^b^ |
| **Hospitalization** |  |
| Hospitalization | €391.20 ^c^ |
| Daytime visits hospital | € 68.65 ^c^ |
| **Vaccination (per vaccinee)** |  |
| Vaccine | € 77.00 |
| Application | € 4.80 |
| Coordination | € 1.65 |

^a^ Consultation fee (Tarief beschikking Nederlandse Zorgautoriteit http://www.nza.nl/9439/10249/41655/tarieven_huisartsen_2008.pdf)

^b^ Costs of medicine as registered in the Dutch formulary were used (http://www.medicijnkosten.nl/)

^c^ Costs of hospitalization were based on the average standard hospitalization costs per inpatient day of general (85%) and academic hospitals (15%) (source: Oostenbrink JB, Bouwmans CAM, Koopmanschap MA, Rutten FFH. Handleiding voor kostenonderzoek; methoden en standaard kostprijzen voor economische evaluaties in de gezondheidszorg. Diemen: College voor zorgverzekeringen, 2004) multiplied by the average duration of hospitalization
